# Unraveling Moral Reasoning in Amyotrophic Lateral Sclerosis: How Emotional Detachment Modifies Moral Judgment

**DOI:** 10.3389/fpsyg.2020.02083

**Published:** 2020-08-21

**Authors:** Chiara Crespi, Gaia Chiara Santi, Alessandra Dodich, Federica Lupo, Lucia Catherine Greco, Tommaso Piccoli, Christian Lunetta, Chiara Cerami

**Affiliations:** ^1^Department of Brain and Behavioral Sciences, University of Pavia, Pavia, Italy; ^2^Istituto Universitario di Studi Superiori, Pavia, Italy; ^3^CeRiN, Center for Mind/Brain Sciences, University of Trento, Rovereto, Italy; ^4^Department of Experimental Biomedicine and Clinical Neurosciences, University of Palermo, Palermo, Italy; ^5^NEuroMuscular Omnicentre, Niguarda Ca’ Granda Hospital, Milan, Italy; ^6^IRCCS Mondino Foundation, Pavia, Italy

**Keywords:** amyotrophic lateral sclerosis, emotion detachment, moral cognition, moral judgment, social cognition

## Abstract

In the last decade, scientific literature provided solid evidence of cognitive deficits in amyotrophic lateral sclerosis (ALS) patients and their effects on end-life choices. However, moral cognition and judgment are still poorly investigated in this population. Here we aimed at evaluating both socio-cognitive and socio-affective components of moral reasoning in a sample of 28 ALS patients. Patients underwent clinical and neuropsychological evaluation including basic cognitive and social cognition measures. Additionally, we administered an experimental task including moral dilemmas, with instrumental and incidental conditions. Patients’ performances were compared with a control group [healthy control (HC)], including 36 age-, gender-, and education-matched healthy subjects. Despite that the judgment pattern was comparable in ALS and HC, patients resulted less prone to carry out a moral transgression compared to HC. Additionally, ALS patients displayed higher levels of moral permissibility and lower emotional arousal, with similar levels of engagement in both instrumental and incidental conditions. Our findings expanded the current literature about cognitive deficits in ALS, showing that in judging moral actions, patients may present non-utilitarian choices and emotion flattening. Such a decision-making profile may have relevant implications in applying moral principles in real-life situations and for the judgment of end-of-life treatments and care in clinical settings.

## Introduction

Amyotrophic lateral sclerosis (ALS) is a progressive neurodegenerative multisystem disorder that selectively targeted the motor system (Hardiman et al., 2017). A variable range of non-motor behavioral and cognitive manifestations affects patients, with a subgroup of ALS developing a frank frontotemporal dementia (FTD) syndrome (Hardiman et al., 2017). Cognitive disorders often characterize the clinical phenotype, with a major involvement of language and executive abilities (Consonni et al., 2013; Beeldman et al., 2016; Strong et al., 2017). Behavioral changes may occur with predominant apathy and depression (Lillo et al., 2011).

In the last decade, scientific literature provided evidence of social cognition deficits in ALS, particularly, in the domains of recognition and processing of emotional materials (Lulé et al., 2005; Papps et al., 2005; Zimmerman et al., 2007; Palmieri et al., 2010; Crespi et al., 2014), affective decision-making (Meier et al., 2010; Girardi et al., 2011), and empathy (Gibbons et al., 2007; Cavallo et al., 2011; Cerami et al., 2014). Notwithstanding such a large literature on cognitive deficits and socio-emotional disorders in ALS, moral cognition has been poorly investigated yet. Research studies on the field mainly investigated ethical implications of end-of-life treatment decisions, supporting the independence between basic cognitive and behavioral impairments and patient decisions on the use of invasive medical devices (Böhm et al., 2016). In this view, the literature suggests the need for longitudinal assessment of decision-making skills to early recognize possible alterations and their effects on end-of-life choices (Khin Khin et al., 2015).

Morality is defined as the set of values and habits adopted by a cultural group in order to orient its social conduct (Moll et al., 2005). Tasks involving moral dilemmas—i.e., situations where an agent cannot fulfill, with his/her choice, all applicable moral requirements—classically measure the patient’s ability to evaluate actions made in respect to a set of virtues (i.e., “moral judgment”). Moral reasoning is defined as the sum of all mental conscious processes that allows the agent to achieve moral judgment (Moll et al., 2005). From the neuroscience perspective, neurocognitive processes underlying moral reasoning primarily encompass two main dimensions: the socio-cognitive and the socio-affective components (Bzdok et al., 2012). In evaluating a moral dilemma, subjects are often required to define the “moral acceptability/permissibility” of the leading character’s behavior (e.g., completely morally unacceptable or completely morally acceptable), referred to the individual’s ability to evaluate the character’s action as tolerable, according to the personal moral values and habits, and to evaluate the experience considering the relative “emotional valence” (e.g., pleasantness/unpleasantness) and “emotional arousal” (e.g., activation/calm). These emotional dimensions represent those parameters that typically account for most of the variance in the final moral judgment (Lotto et al., 2014).

A recent paper on moral judgment in ALS and behavioral variant of frontotemporal dementia (bvFTD) (Semler et al., 2019) proved comparable poor patient’s attitude in evaluating a moral situation in both neurodegenerative conditions. In this study, experimental assessment included the Moral Competence Test, the Ethics Position Questionnaire, and the Idler Index of Religiosity to respectively test moral competence, moral position, and religiosity. Impairment in moral judgment was regardless of ethic and religiosity scores and did not correlate with basic cognitive performances (Semler et al., 2019).

The influence of affective parameters on moral judgment processes in ALS patients is a matter of debate. No detailed information about the socio-affective dimension of moral reasoning was available yet. Some studies on other neurodegenerative patients (e.g., FTD) investigated the relationship between moral judgment, basic and social cognition abilities (Mendez et al., 2005; Mendez and Shapira, 2009; Gleichgerrcht et al., 2011) showing low performance, compared to control subjects, in a subtask requiring an immediate emotionally driven moral judgment (Mendez et al., 2005). The role of affective and emotional processes on moral decision-making in bvFTD is also supported by results of Gleichgerrcht et al. (2011).

In this view, our research study was aimed at investigating how affective and emotional dimensions influence the decision-making process of ALS patients in a moral dilemmas task.

## Materials and Methods

### Subjects

Twenty-eight patients with a diagnosis of either probable or definite ALS (Brooks et al., 2000) (25 males; mean age = 57.79 ± 11.72 years; mean education = 11.50 ± 4.78 years; mean disease duration from the onset = 1.84 ± 1.47 years) were enrolled (Table 1) for the present study. According to the clinical presentation, ALS patients were classified as 19 spinal and nine bulbar onsets. We excluded patients with global cognitive deficits, as revealed by a mini mental state examination (MMSE) raw score < 23 (Duchesne et al., 2005; Diniz et al., 2007; Mitchell, 2017), or comorbid psychiatric disorders potentially interfering with cognitive functioning, as well as patients with respiratory disorders (forced vital capacity < 70% of predicted capacity), severe dysarthria, and communication difficulties potentially invalidating the administration and/or interpretation of neuropsychological assessment.

**TABLE 1:** Demographic, clinical, and neuropsychological features of the sample.

(A) Demographic and clinical features			

	ALS	HC	Statistics
*Female/Male ratio*	3/25	11/25	χ^2^(1) = 0.057
*Age in years (mean ± SD)*	57.79 ± 11.72	52.97 ± 14.03	*t*(62) = 0.603
*Education in years (mean ± SD)*	11.50 ± 4.79	9.97 ± 3.17	*t*(62) = 0.075
*Disease duration in years (mean ± SD)*	1.84 ± 1.47	–	–
*Age at the onset (mean ± SD)*	55.15 ± 11.54	–	–
*ALS-FRSr (mean ± SD)*	29.80 ± 7.18	–	–

**(B) Cognitive and behavioral assessment**			

		% out of cutoff score*	% borderline score*

*Mini mental state examination (mean ± SD)*	26.29 ± 2.44	(6/28) 21.4%	–
*ECAS-global score (mean ± SD)*	106.54 ± 12.49	(4/28) 14.28%	(1/28) 3.57%
*ECAS-specific (mean ± SD)*	77.72 ± 11.09	(4/28) 14.28%	(3/28) 10.71%
*ECAS-non-specific (mean ± SD)*	28.61 ± 3.0	(1/28) 3.57%	(3/28) 10.71%
*SET global score (mean ± SD)*	12.57 ± 3.78	(5/28) 17.9%	(3/28) 10.7%
*SET emotion attribution condition (mean ± SD)*	4.50 ± 1.36	(2/28) 7.1%	(5/28) 17.9%
*SET intention attribution condition (mean ± SD)*	4.24 ± 1.60	(4/28) 14.3%	(6/28) 21.4%
*SET causal inference condition (mean ± SD)*	3.94 ± 1.60	(5/28) 17.9%	(4/28) 14.3%
*Ek-60F global score (mean ± SD)*	46.71 ± 6.70	(4/28) 14.3%	(2/28) 7.1%

*The table reports, **(A)** demographic information for patients and controls, as well as clinical characteristics of the patient group. **(B)** Illustrates scores obtained from the cognitive and behavioral assessment in patients, reporting mean and standard deviation (SD), the proportion of patients scoring out of the cutoff score and that of patients obtaining a borderline score. ALS, amyotrophic lateral sclerosis; HC, healthy control; ALS-FRSr, ALS functional rating scale revised; ECAS, Edinburgh cognitive and behavioral ALS screen battery; SET, story-based empathy task; Ek-60F, Ekman 60 Faces test. ^∗^percentage of patients obtaining an impaired/borderline performance was computed according to the Italian normative values.*

Thirty-six age-, gender-, and education-matched healthy controls (HCs; 25 males; mean age = 52.97 ± 14.03 years; mean education = 9.97 ± 3.17 years) were recruited at local senior community centers. They underwent a clinical interview, a neurologic examination, and a brief neuropsychological assessment in order to test cognitive efficiency. Medical history positive for neuropsychiatric disorders, positive neurologic examination, MMSE raw score <28 (Measso et al., 1993; Crespi et al., 2014, 2016; Van Patten et al., 2019), as well as verbal and visuospatial delayed memory performances (i.e., Rey Auditory Verbal Learning test, Rey Figure Recall task) below 25th percentile according to the Italian normative values, were considered as exclusion criteria for HC enrollment.

### Basic Cognitive and Social Assessment of Amyotrophic Lateral Sclerosis Patients

Basic cognitive functioning in patients was evaluated with the Italian version of the Edinburgh Cognitive and Behavioral ALS Screen (ECAS) battery. This is a widely used multi-domain brief assessment designed to evaluate a range of cognitive functions typically affected in ALS (i.e., ALS-Specific domains: executive functions, social cognition, fluency, and language) (Poletti et al., 2016). In addition to executive (reverse digit span, alternation, and sentence completion tasks), social cognition (Yoni task), fluency (verbal fluency tasks for words beginning with the letter “S” and for four-letter words starting with the letter “C”), and language (naming, comprehension, and spelling) tasks, ECAS explored also cognitive domains not typically affected in ALS but common in pathological aging [ALS Non-specific domains: memory (immediate recall and delayed recognition) and visuospatial (dot counting, cube counting, and number location)] (Poletti et al., 2016). Neuropsychological assessment also included the MMSE (Folstein et al., 1975) to assess global cognitive efficiency. Primary caregivers were asked to complete the frontal behavioral inventory (FBI) (Kertesz et al., 1997; Alberici et al., 2007) to assess behavioral changes in patients.

A brief social cognition battery exploring basic emotion recognition and emotion and intention attribution in others was also administered including the Ekman-60-Faces test (Ek-60F) (Dodich et al., 2014) and the Story-based Empathy Task (SET) (Dodich et al., 2015). The EK-60F consists of 60 b/w pictures from the Ekman and Friesen series of pictures of facial affect. Pictures depict the faces of 10 actors, each displaying one of the six basic emotions (i.e., happiness, sadness, anger, fear, surprise, and disgust). The SET is a non-verbal cartoon task that consists of two main experimental conditions, i.e., identifying intentions (SET-IA) and emotional states (SET-EA), plus a control condition entailing the inference of causality reaction based on the knowledge of the physical properties of objects and human bodies (SET-CI). These tasks have been proved to be sensitive on ALS patients (Girardi et al., 2011; Crespi et al., 2014).

All subjects gave informed consent to the experimental procedure, which was approved by the local ethics committee.

See Table 1 for details on demographic variables.

### Experimental Moral Task

The task of moral dilemmas includes eight scenarios derived from Lotto et al. (2014). Each dilemma was presented as text in two phases. The first phase described the scenario in which different kinds of threats were going to cause death to a group of people. The second phase described a hypothetical resolution in which the participant, identifying him/herself as the main character, can choose to kill or not one individual to save the others, who otherwise would have died. Dilemmas were subgrouped into two conditions: “instrumental” (i.e., the death of one person is a mean to save more people) and “incidental” (i.e., the death of one person is a foreseen but unintended consequence of the action aimed at saving more people). Additionally, half of dilemmas involved directly the main character because its life was at risk (i.e., self-involvement). See Supplementary Table S1 for stimulus examples.

Participants were asked to indicate whether they would do the proposed action. Then, they were asked to judge how morally acceptable was the resolution (0 = not at all, 7 = completely), to attribute an emotional valence to the moral action (0 = totally unpleasant, 8 = totally pleasant), and to rate the emotional arousal (0 = totally calm, 8 = totally involved). Outcome variables were (i) the rate of yes/no responses to the proposed resolution (i.e., moral judgment); the rating of (ii) moral acceptability, (iii) emotional valence, and (iv) emotional arousal experienced during the decision-making process.

### Statistical Analyses

We explored patients’ performances at the moral dilemmas task in comparison to the control group. Preliminarily, we explored the distribution for each variable with the Kolmogorov–Smirnov test. Although some variables did not show a normal distribution, we analyzed data by testing parametric models as well (one-way ANOVA, mixed ANOVA) according to the results from Blanca et al. (2017).

First, Chi-Square Independence test was used to analyze group differences in rate of yes/no responses at moral dilemmas for the diverse task conditions of both type of dilemma (instrumental or incidental conditions) and personal involvement factors (self-involvement or no self-involvement conditions). We additionally performed one-way ANOVA on percentages of yes/no responses to provide a better description of the pattern of utilitarian responses in ALS vs. HC.

Mixed-ANOVA models were then computed to detect differences in the rating of moral permissibility, emotional valence, and emotional arousal experienced during the decision-making process, considering the type of dilemma and the personal involvement as within-subject variables and the group (ALS and HC) as a between-subject variable.

Analyses were conducted using IBM SPSS Statistics for Windows v24.0 (IBM Corporation, Armonk, NY, United States).

## Results

### Basic Cognitive and Social Impairments in Amyotrophic Lateral Sclerosis Patients

Six out of the 28 patients (21.4%) had an impaired performance on the MMSE, scoring under the cutoff. Four out of 28 ALS patients (14.28%) showed significant impairments in the ECAS global score, and one patient had a borderline score. Four of these five patients with impaired and/or borderline ECAS global score presented impaired and/or reduced performance at ECAS ALS-specific cognitive domains and at social cognition tests. Seven additional patients (25%) showed isolated impaired or borderline scores at the ECAS ALS-specific cognitive domains. Only one patient showed significant impairments at the ECAS ALS non-specific cognitive domains, while three others obtained a borderline performance at ALS non-specific tasks. Impairments of Ek-60F and SET occurred either isolated or combined in patients. Global EK-60F or SET scores were impaired or borderline in half of the patient sample (50%, 14/28). FBI was completed by 20/28 caregivers, showing mild-to-moderate behavioral changes in patients. Increased negative (6.42 ± 6.38) and positive (3.11 ± 3.17) symptoms were both reported, with a prevalence of symptoms as apathy, emotional flatness, loss of insight, and inflexibility. According to the criteria by Strong et al. (2017), six patients (21.5%) were classified as pure ALS, six (21.5%) as ALSci, four (14%) as ALSbi, and four (14%) as ALScbi. Eight patients (29%) were not classifiable as their caregivers did not complete the FBI.

See Table 1 for further details.

### Moral Cognition Disorders in Amyotrophic Lateral Sclerosis Patients Compared to Healthy Controls

Overall, the ALS sample showed impairments in moral judgment ability. Chi-Square Independence test highlighted that the global rate of yes/no response in resolution of moral dilemmas was significantly different in ALS compared to HC (Incidental dilemmas: χ^2^ = 36.70, *p* < 0.001; Instrumental dilemmas: χ^2^ = 14.65, *p* = 0.005; No self-involvement: χ^2^ = 38.39, *p* < 0.001; Self-involvement: χ^2^ = 18.25, *p* < 0.001). These results were confirmed by one-way ANOVA results, with ALS patients showing significantly lower percentages of utilitarian responses than HC in each condition, both factors considered [i.e., type of dilemma: incidental: *F*(1,62) = 38.16, *p* < 0.001; instrumental: *F*(1,62) = 3.81, *p* = 0.05; personal involvement: no self-involvement: *F*(1,62) = 20.36, *p* < 0.001; self-involvement: *F*(1,62) = 13.02, *p* = 0.001].

**FIGURE 1:**
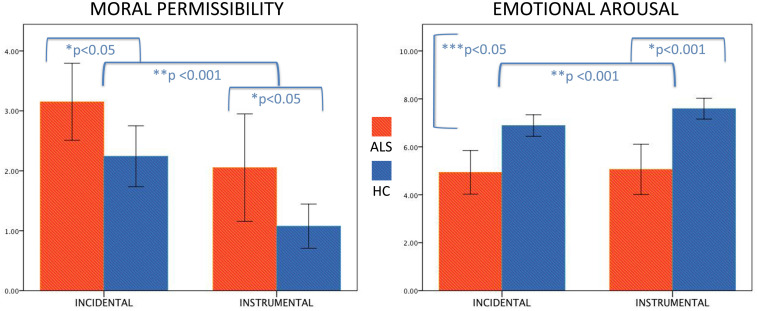
Significant differences in moral permissibility and emotional arousal in amyotrophic lateral sclerosis (ALS) patients compared to healthy controls (HCs). The figure depicts, on the **left**, the significant group (*) and type of dilemma (**) effects for the moral permissibility variable, showing significantly higher ratings in ALS patients than HC and higher ratings for incidental compared to instrumental dilemmas in both groups. On the **right**, we report significant group (*), type of dilemma (**) and group × type of dilemma interaction (***) effects for the emotional arousal variable, highlighting lower ratings in ALS patients than HC and higher ratings for instrumental than incidental dilemmas in both groups, with HC showing a greater difference between instrumental and incidental conditions than ALS patients.

Concerning the judgment of moral permissibility, the result of a 2 × 2 (type of dilemma condition × group) mixed ANOVA showed a significant effect of both group [*F*(1,62) = 6.1, *p* = 0.016] and type of dilemma [*F*(1,62) = 46.58, *p* < 0.001] with no interaction effect, indicating significantly higher ratings in ALS patients than HC; higher ratings for incidental compared to instrumental dilemmas characterized both groups (Figure 1). Results from personal involvement × group mixed ANOVA highlighted a significant effect of group [*F*(1,62) = 6.1, *p* = 0.016], with ALS patients showing higher ratings than HC. We did not find a significant effect of personal involvement nor an interaction effect with group.

About ratings of emotional valence, the result of a 2 × 2 (type of dilemma condition × group) mixed ANOVA showed a significant effect of the type of dilemma [*F*(1,62) = 19.96, *p* < 0.001], describing low emotional valence for instrumental dilemmas compared to incidental ones in both groups, while we did not observe significant group and interaction effects. We found no significant effects in personal involvement × group mixed ANOVA.

About evaluation of emotional arousal, the result of a 2 × 2 (type of dilemma condition × group) mixed ANOVA showed a significant effect of both group [*F*(1,62) = 22.33, *p* < 0.001] and type of dilemma [*F*(1,62) = 13.16, *p* = 0.001], as well as a significant interaction effect [*F*(1,62) = 6.403, *p* = 0.014], revealing lower ratings in ALS patients than HC and higher ratings for instrumental than incidental dilemmas in both groups, with HC showing a greater difference between instrumental and incidental conditions than ALS (Figure 1). Results from personal involvement × group mixed ANOVA highlighted a significant effect of group [*F*(1,62) = 22.33, *p* < 0.001] with ALS patients showing lower ratings than HC. We did not find a significant effect of the personal involvement nor an interaction effect with group. See Table 2 for further details.

**TABLE 2:** Performance at moral judgment task in patients and healthy controls.

A	*Type of Dilemma*				*Significant results*
	*Incidental*		*Instrumental*		
*Group*	ALS	HC	ALS	HC	
*Affirmative response%*	14.28%	88.8%	7.14%	47.22%	Group difference (*Incidental*): χ^2^(4) = 36.70, *p* < 0.001 Group difference (*Instrumental*): χ^2^(4) = 14.65, *p* = 0.005
*Moral permissibility*	3.15 ± 1.65	2.24 ± 1.50	2.05 ± 2.31	1.07 ± 1.08	Group effect: *F*(1,62) = 6.09, *p* < 0.001 Type of dilemma effect: *F*(1,62) = 46.58, *p* < 0.001
*Emotional arousal*	4.93 ± 2.34	6.88 ± 1.32	5.06 ± 2.70	7.59 ± 1.28	Group effect: *F*(1,62) = 22.33, *p* < 0.001 Type of dilemma effect: *F*(1,62) = 13.162, *p* = 0.001 Interaction effect: *F*(1,62) = 6.40, *p* = 0.014
*Emotional valence*	2.20 ± 1.40	2.22 ± 1.06	2.00 ± 1.20	1.74 ± 0.79	Type of dilemma effect: *F*(1,62) = 19.96, *p* < 0.001

**B**	***Involvement***				
	***Self***		***No self***		
*Group*	**ALS**	**HC**	**ALS**	**HC**	

*Affirmative response%*	10.71%	61.11%	10.71%	86.11%	Group difference (*Self*): χ^2^(4) = 18.25, *p* = 0.001 Group difference (*No self*): χ^2^(4) = 38.38, *p* < 0.001
*Moral permissibility*	2.48 ± 1.84	1.73 ± 1.39	2.72 ± 2.06	1.58 ± 1.08	Group effect: *F*(1,62) = 6.09, *p* < 0.001
*Emotional arousal*	5.03 ± 2.60	7.12 ± 1.19	4.96 ± 2.45	7.35 ± 1.37	Group effect: *F*(1,62) = 22.33, *p* < 0.001
*Emotional valence*	2.16 ± 1.37	2.06 ± 0.88	2.04 ± 1.23	1.90 ± 1.02	**–**

*The table reports performances at the moral judgment task and significant results obtained from the analysis of variables of interest (i.e., proportion of affirmative responses, moral permissibility, emotional arousal, and emotional valence). While **(A)** shows results from mixed ANOVA considering the type of dilemma (incidental vs. instrumental) as between variable, **(B)** reports results from mixed ANOVA considering the personal involvement (self vs. no self-involvement) as between variables. ALS, amyotrophic lateral sclerosis; HC, healthy control.*

## Discussion

Moral cognition is still a poorly investigated domain in ALS. Although previous results suggested that ALS patients presented deficits in integrating their moral values into situational conditions, even if their knowledge of rules and ethics dogma is preserved (Semler et al., 2019), the impact of changes in affective processing on decision-making, and specifically related to the moral judgment, has not been estimated yet.

In the present study, we explored moral cognition in ALS patients using one of the most reliable moral tasks (Lotto et al., 2014) and extensively applied on neuropsychiatric populations (Pletti et al., 2017) and healthy aging (McNair et al., 2018), aiming at assessing moral judgment controlling for different variables (i.e., moral acceptability, emotional valence, and arousal).

Our findings provided evidence of different moral judgment behaviors in patients compared to HC. In detail, ALS showed significantly lower yes/no rate of responses both in incidental and instrumental conditions, also considering dilemmas on the basis of personal involvement. At the same time, patients displayed higher levels of moral permissibility than HC, reflecting an overall tendency to judge as more acceptable actions that they would not actually perform. Such a performance may reflect a defective evaluation of the consequences of the moral transgression committed in the scenario. Indeed, patients showed less utilitarian behaviors and more emotion-based evaluations compared to healthy subjects. Although moral judgment is considered to occur quickly as an intuitive automatic response, based on deontological principles rather than being a conscious and rational reasoning process that follows a careful evaluation of the moral situation (Haidt, 2001), the definition of the acceptability of moral actions entails the engagement of specific decision-making competences to calculate the preferable solution (Bretz and Sun, 2018). Rational calculation of moral actions usually results in utilitarian solutions (the greatest good for the greatest number), while emotion-based evaluations imply taking non-utilitarian behaviors, as seen in our patient sample. Preserved social cognition skills are thus crucial to maintain the best efficiency in affective decision-making processes and in moral cognition. The impairments of decision-making processing of moral actions ascribe ALS to a more extensive social cognition dysfunction (Lulé et al., 2005; Papps et al., 2005; Zimmerman et al., 2007; Meier et al., 2010; Palmieri et al., 2010; Cavallo et al., 2011; Girardi et al., 2011; Cerami et al., 2014; Crespi et al., 2014), as also proved by the neuropsychological performances of our sample. About a third of the patient group displayed in fact impaired or borderline performances at emotion recognition and socio-emotional processing tasks. Impairments of such a multifaceted cognitive domain may be underestimated by ECAS battery alone. According to this, an in-depth assessment of social cognition performances in ALS should be systematically promoted.

Our evidence on the emotion arousal subtask of moral dilemmas further confirmed what was previously stated. HC showed higher emotional arousal ratings for both incidental and instrumental conditions compared to ALS patients, with a greater arousal for incidental vs. instrumental dilemmas. Patients showed similar levels of emotional engagement in both conditions. These findings are suggestive of an incipient emotional blunting, which can be clinically observed in a proportion of ALS patients, also at early stages (Phukan et al., 2007; Strong et al., 2009), and that can interfere with the ability to modulate the application of moral principles in a consistent and nuanced manner to the different social situations.

From a cognitive neuroscience perspective, brain substrates underlying cognitive and affective facets of moral cognition (e.g., temporoparietal junction, medial prefrontal cortex, middle temporal gyrus) are common to nodes included in brain networks of both theory of mind (ToM) and empathy (Bzdok et al., 2012). In particular, brain atrophy and changes in white matter integrity in structures supporting socio-affective skills (i.e., ventromedial prefrontal and temporo-limbic cortex and right ventral associative bundles) have proven to occur early in ALS (Cerami et al., 2014; Crespi et al., 2014, 2016). These findings are in line with neuropsychological findings of the present and previous studies indicating reduced emotional engagement, emotion recognition, and attribution in ALS patients (Lulé et al., 2005; Papps et al., 2005; Zimmerman et al., 2007; Palmieri et al., 2010; Girardi et al., 2011; Crespi et al., 2014). Indeed, significant differences in patients compared to controls in the socio-affective component of moral reasoning might reflect anatomo-functional alterations in brain regions normally recruited for the elaboration of socio-emotional stimuli and thus impacting on judgments about moral permissibility of fictitious scenarios (Bzdok et al., 2012).

## Conclusion

Although the small sample size, the cross-sectional design, and the lack of neuroimaging markers of moral changes limit the generalizability of our findings, we expand current literature (Semler et al., 2019) suggesting for the first time that the presence of deficits in emotion recognition and mental state attribution abilities, and emotional flattening (socio-affective component of moral decision-making) may influence the attitude in evaluating a moral situation (socio-cognitive component of moral decision-making) in ALS patients. Moreover, this study contributes to define the extent and boundaries of the frontotemporal cognitive syndrome in ALS.

Besides its theoretical interest, deficits in the judgment of moral actions entail crucial practical implications for the management of neurological patients and should be appropriately taken into account in the evaluation of therapeutic approaches and critical life choices. Further studies based on clinical settings are needed to confirm our data and estimate in ALS the weight of moral judgment alterations and their relationship with the quality of life and socio-affective disorders at the individual level, particularly with regard to gender effect and disease subtype. Overall, the variable vulnerability to social and non-social cognitive changes observed in ALS patients indicates that larger longitudinal studies are also needed to estimate the impact of moral cognition impairments in real-life situations (e.g., end-life decisions).

## Data Availability Statement

The raw data supporting the conclusions of this article will be made available by the authors, without undue reservation.

## Ethics Statement

The studies involving human participants were reviewed and approved by the San Raffaele Hospital Ethics Committee. The patients/participants provided their written informed consent to participate in this study.

## Author Contributions

CCr: conceptualization, formal analysis, writing – original draft, and visualization. GS: investigation, formal analysis, and writing – reviewing and editing. AD: investigation and writing – reviewing and editing. FL and LG: investigation and resources. TP and CL: investigation and supervision. CCe: conceptualization, project administration, supervision, investigation, data curation, and writing – reviewing and editing. All authors contributed to the article and approved the submitted version.

## Conflict of Interest

The authors declare that the research was conducted in the absence of any commercial or financial relationships that could be construed as a potential conflict of interest.

## References

[B1] Alberici A. Geroldi C. Cotelli M. Adorni A. Calabria M. Rossi G. (2007). The Frontal Behavioural Inventory (Italian version) differentiates frontotemporal lobar degeneration variants from Alzheimer’s disease. *Neurol. Sci.* 28 80–86. 10.1007/s10072-007-0791-3 17464470

[B2] Beeldman E. Raaphorst J. Klein Twennaar M. de Visser M. Schmand B. A. de Haan R. J. (2016). The cognitive profile of ALS: a systematic review and meta-analysis update. *J. Neurol. Neurosurg. Psychiatry* 87 611–619. 10.1136/jnnp-2015-310734 26283685

[B3] Blanca M. Alarcón R. Arnau J. Bono R. Bendayan R. (2017). Non-normal data: is ANOVA still a valid option? *Psicothema* 29 552–557. 29048317 10.7334/psicothema2016.383

[B4] Böhm S. Aho-Özhan H. E. A. Keller J. Dorst J. Uttner I. Ludolph A. C. (2016). Medical decisions are independent of cognitive impairment in amyotrophic lateral sclerosis. *Neurology* 87 1737–1738. 10.1212/WNL.0000000000003232 27664982

[B5] Bretz S. Sun R. (2018). Two models of moral judgment. *Cogn. Sci.* 42 4–37. 10.1111/cogs.12517 28685842

[B6] Brooks B. R. Miller R. G. Swash M. Munsat T. L. (2000). El Escorial revisited: revised criteria for the diagnosis of amyotrophic lateral sclerosis. *Amyotroph. Lateral Scler. Other Motor Neuron Disord.* 1 293–299. 10.1080/146608200300079536 11464847

[B7] Bzdok D. Schilbach L. Vogeley K. Schneider K. Laird A. R. Langner R. (2012). Parsing the neural correlates of moral cognition: ALE meta-analysis on morality, theory of mind, and empathy. *Brain Struct. Funct.* 217 783–796. 10.1007/s00429-012-0380-y 22270812 PMC3445793

[B8] Cavallo M. Adenzato M. MacPherson S. E. Karwig G. Enrici I. Abrahams S. (2011). Evidence of social understanding impairment in patients with amyotrophic lateral sclerosis. *PLoS One* 6:e25948. 10.1371/journal.pone.0025948 21998727 PMC3187828

[B9] Cerami C. Dodich A. Canessa N. Crespi C. Iannaccone S. Corbo M. (2014). Emotional empathy in amyotrophic lateral sclerosis: a behavioural and voxel-based morphometry study. *Amyotroph. Lateral Scler. Frontotemporal Degener.* 15 21–29. 10.3109/21678421.2013.785568 23586919

[B10] Consonni M. Iannaccone S. Cerami C. Frasson P. Lacerenza M. Lunetta C. (2013). The cognitive and behavioural profile of amyotrophic lateral sclerosis: application of the consensus criteria. *Behav. Neurol.* 27 143–153. 10.3233/BEN-2012-110202 23001631 PMC5215720

[B11] Crespi C. Cerami C. Dodich A. Canessa N. Arpone M. Iannaccone S. (2014). Microstructural white matter correlates of emotion recognition impairment in Amyotrophic Lateral Sclerosis. *Cortex* 53 1–8. 10.1016/j.cortex.2014.01.002 24534360

[B12] Crespi C. Cerami C. Dodich A. Canessa N. Iannaccone S. Corbo M. (2016). Microstructural correlates of emotional attribution impairment in non-demented patients with amyotrophic lateral sclerosis. *PLoS One* 11:e0161034. 10.1371/journal.pone.0161034 27513746 PMC4981464

[B13] Diniz B. S. Yassuda M. S. Nunes P. V. Radanovic M. Forlenza O. V. (2007). Mini-mental state examination performance in mild cognitive impairment subtypes. *Int. Psychogeriatr.* 19 647–656. 10.1017/s104161020700542x 17502007

[B14] Dodich A. Cerami C. Canessa N. Crespi C. Iannaccone S. Marcone A. (2015). A novel task assessing intention and emotion attribution: Italian standardization and normative data of the Story-based Empathy Task. *Neurol. Sci.* 36 1907–1912. 10.1007/s10072-015-2281-3 26072203

[B15] Dodich A. Cerami C. Canessa N. Crespi C. Marcone A. Arpone M. (2014). Emotion recognition from facial expressions: a normative study of the Ekman 60-Faces Test in the Italian population. *Neurol. Sci.* 35 1015–1021. 10.1007/s10072-014-1631-x 24442557

[B16] Duchesne S. Caroli A. Geroldi C. Frisoni G. B. Collins D. L. (2005). “Predicting clinical variable from MRI features: application to MMSE in MCI,” in *International Conference on Medical Image Computing and Computer-Assisted Intervention*, eds Duncan J. S. Gerig G. (Berlin: Springer), 392–399. 10.1007/11566465_49 16685870

[B17] Folstein M. F. Folstein S. E. McHug H. P. R. (1975). “Mini-mental state”: a practical method for grading the cognitive state of patients for the clinician. *J. Psychiatr. Res.* 12 189–198. 10.1016/0022-3956(75)90026-6 1202204

[B18] Gibbons Z. C. Snowden J. S. Thompson J. C. Happe F. Richardson A. Neary D. (2007). Inferring thought and action in motor neurone disease. *Neuropsychologia* 45 1196–1207. 10.1016/j.neuropsychologia.2006.10.008 17118410

[B19] Girardi A. MacPherson S. E. Abrahams S. (2011). Deficits in emotional and social cognition in amyotrophic lateral sclerosis. *Neuropsychology* 25 53–65. 10.1037/a0020357 20919762

[B20] Gleichgerrcht E. Torralva T. Roca M. Pose M. Manes F. (2011). The role of social cognition in moral judgment in frontotemporal dementia. *Soc. Neurosci.* 6 113–122. 10.1080/17470919.2010.506751 20706963

[B21] Haidt J. (2001). The emotional dog and its rational tail: a social intuitionist approach to moral judgment. *Psychol. Rev.* 108 814–834. 10.1037/0033-295x.108.4.814 11699120

[B22] Hardiman O. Al-Chalabi A. Chio A. Corr E. M. Logroscino G. Robberecht W. (2017). Amyotrophic lateral sclerosis. *Nat. Rev. Dis. Primers* 3:17071. 10.1038/nrdp.2017.71 28980624

[B23] Kertesz A. Davidson W. Fox H. (1997). Frontal behavioral inventory: diagnostic criteria for frontal lobe dementia. *Can. J. Neurol. Sci.* 24 29–36. 10.1017/s0317167100021053 9043744

[B24] Khin Khin E. Minor D. Holloway A. Pelleg A. (2015). Decisional capacity in amyotrophic lateral sclerosis. *J. Am. Acad. Psychiatry Law* 43 210–217. 26071511

[B25] Lillo P. Mioshi E. Zoing M. C. Kiernan M. C. Hodges J. R. (2011). How common are behavioural changes in amyotrophic lateral sclerosis? *Amyotroph. Lateral Scler.* 12 45–51. 10.3109/17482968.2010.520718 20849323

[B26] Lotto L. Manfrinati A. Sarlo M. (2014). A new set of moral dilemmas: norms for moral acceptability, decision times, and emotional salience. *J. Behav. Decis. Mak.* 27 57–65. 10.1002/bdm.1782

[B27] Lulé D. Kurt A. Jürgens R. Kassubek J. Diekmann V. Kraft E. (2005). Emotional responding in amyotrophic lateral sclerosis. *J. Neurol.* 252 1517–1524. 15977000 10.1007/s00415-005-0907-8

[B28] McNair S. Okan Y. Hadjichristidis C. de Bruin W. B. (2018). Age differences in moral judgment: older adults are more deontological than younger adults. *J. Behav. Decis. Mak.* 32 47–60. 10.1002/bdm.2086

[B29] Measso G. Cavarzeran F. Zappalà G. Lebowitz B. D. Crook T. H. Pirozzolo F. J. (1993). The mini-mental state examination: normative study of an Italian random sample. *Dev. Neuropsychol.* 9 77–85. 10.1080/87565649109540545

[B30] Meier S. L. Charleston A. J. Tippett L. J. (2010). Cognitive and behavioural deficits associated with the orbitomedial prefrontal cortex in amyotrophic lateral sclerosis. *Brain* 133 3444–3457. 10.1093/brain/awq254 20889583

[B31] Mendez M. F. Anderson E. Shapira J. S. (2005). An investigation of moral judgement in frontotemporal dementia. *Cogn. Behav. Neurol.* 18 193–197. 10.1097/01.wnn.0000191292.17964.bb 16340391

[B32] Mendez M. F. Shapira J. S. (2009). Altered emotional morality in frontotemporal dementia. *Cogn. Neuropsychiatry* 14 165–179. 10.1080/13546800902924122 19499384

[B33] Mitchell A. J. (2017). “The Mini-Mental State Examination (MMSE): update on its diagnostic accuracy and clinical utility for cognitive disorders,” in *Cognitive Screening Instruments*, ed. Larner A. J. (Cham: Springer), 37–48. 10.1007/978-3-319-44775-9_3

[B34] Moll J. Zahn R. de Oliveira-Souza R. Krueger F. Grafman J. (2005). Opinion: the neural basis of human moral cognition. *Nat. Rev. Neurosci.* 6 799–809. 10.1038/nrn1768 16276356

[B35] Palmieri A. Naccarato M. Abrahams S. Bonato M. D’Ascenzo C. Balestreri S. (2010). Right hemisphere dysfunction and emotional processing in ALS: an fMRI study. *J. Neurol.* 257 1970–1978. 10.1007/s00415-010-5640-2 20593194

[B36] Papps B. Abrahams S. Wicks P. Leigh P. N. Goldstein L. H. (2005). Changes in memory for emotional material in amyotrophic lateral sclerosis (ALS). *Neuropsychologia* 43 1107–1114. 10.1016/j.neuropsychologia.2004.11.027 15817168

[B37] Phukan J. Pender N. P. Hardiman O. (2007). Cognitive impairment in amyotrophic lateral sclerosis. *Lancet Neurol.* 6 994–1003. 17945153 10.1016/S1474-4422(07)70265-X

[B38] Pletti C. Lotto L. Buodo G. Sarlo M. (2017). It’s immoral, but I’d do it! Psychopathy traits affect decision-making in sacrificial dilemmas and in everyday moral situations. *Br. J. Psychol.* 108 351–368. 10.1111/bjop.12205 27370950

[B39] Poletti B. Solca F. Carelli L. Madotto F. Lafronza A. Faini A. (2016). The validation of the Italian Edinburgh Cognitive and Behavioural ALS Screen (ECAS). *Amyotroph. Lateral Scler. Frontotemporal Degener.* 17 489–498. 10.1080/21678421.2016.1183679 27219526

[B40] Semler E. Petersdorff L. Anderl-Straub S. Böhm S. Lulé D. Fangerau H. (2019). Moral judgment in patients with behavioral variant of frontotemporal dementia and amyotrophic lateral sclerosis: no impairment of the moral position, but rather its execution. *Amyotroph. Lateral Scler Frontotemporal Degener.* 20 12–18. 10.1080/21678421.2018.1534972 30513214

[B41] Strong M. J. Abrahams S. Goldstein L. H. Woolley S. Mclaughlin P. Snowden J. (2017). Amyotrophic lateral sclerosis - frontotemporal spectrum disorder (ALS-FTSD): revised diagnostic criteria. *Amyotroph. Lateral Scler. Frontotemporal Degener.* 18 153–174. 10.1080/21678421.2016.1267768 28054827 PMC7409990

[B42] Strong M. J. Grace G. M. Freedman M. Lomen-Hoerth C. Woolley S. Goldstein L. H. (2009). Consensus criteria for the diagnosis of frontotemporal cognitive and behavioural syndromes in amyotrophic lateral sclerosis. *Amyotroph. Lateral. Scler.* 10 131–146. 10.1080/17482960802654364 19462523

[B43] Van Patten R. Britton K. Tremont G. (2019). Comparing the Mini-Mental State Examination and the modified Mini-Mental State Examination in the detection of mild cognitive impairment in older adults. *Int. Psychogeriatr.* 31 693–701. 10.1017/s1041610218001023 30021667

[B44] Zimmerman E. K. Zachary Simmons M. D. Barrett A. M. (2007). Emotional perception deficits in amyotrophic lateral sclerosis. *Cogn. Behav. Neurol.* 20 79–82. 10.1097/wnn.0b013e31804c700b 17558250 PMC1905862

