# Supplementary material for: Unraveling Moral Reasoning in Amyotrophic Lateral Sclerosis: How Emotional Detachment Modifies Moral Judgment
Source: Front Psychol. 2020 Aug 21;11:2083. doi: 10.3389/fpsyg.2020.02083 (PMC7471658; doi:10.3389/fpsyg.2020.02083)
Supplement: Supplementary file 1 [file Table_1.DOCX]

**SUPPLEMENTARY MATERIAL**

**Supplementary Table 1.** The table reports examples of stimuli (a stimulus per condition and per type) from the moral dilemma task.

| **Dilemma** | **Scenario** | **Resolution** |
| --- | --- | --- |
| *INSTRUMENTAL / Self-involvement* | You are the fourth of five hikers in a mountain climb. The leader just secured his rope when the second hiker starts sliding down dragging all the others with him. The group, including you, falls for tens of meters and stops on the edge of a cliff. You are too many and too heavy, and the rope will not hold all that weight. | To lighten the weight, you cut the rope between you and the last hiker. You know he will fall and die, but you and the other two hikers will be safe. |
| *INSTRUMENTAL / Other-involvement* | You are the supervisor of a team working on a very big lift. Six workers are operating in the elevator shaft. You and a colleague are at the top floor, in the motor room. Suddenly the winch breaks, and the cabin starts falling. | You push your colleague into the gears, so that his body will stop the motor and the cabin will not fall. You know he will die, but the other six workers will be safe. |
| *INCIDENTAL / Self-involvement* | You are a taxi driver and you are taking two passengers at night. It has been snowing for a couple of hours and the road are dangerously iced. You turn into an alley and suddenly you find an upset truck in the middle of the road. You start breaking but the brakes do not respond and the wheels start spinning loosing grip. | You briskly turn the car towards the side of the road. You see a pedestrian, and you know he will die hit by the car, but you and the two passengers will be safe. |
| *INCIDENTAL / Other-involvement* | You are a building worker who is maneuvering a crane on a building site. You have just started your day on the site, when you realize that the cable of the crane is about to break. Attached to the cable is an enormous steel beam that is directly above a crew of six who are working on the outside of a building in construction. | You move the arm of the crane a short distance to another area of the site. You know that there is a worker there who will be crushed by the steel beam and will die, but the other six workers will be unhurt. |
